# Reliability, Responsiveness, and Validity of the Visual Analog Fatigue Scale to Measure Exertion Fatigue in People with Chronic Stroke: A Preliminary Study

**DOI:** 10.4061/2010/412964

**Published:** 2010-05-16

**Authors:** Benjamin Y. Tseng, Byron J. Gajewski, Patricia M. Kluding

**Affiliations:** ^1^Department of Physical Therapy, and Rehabilitation Science, The University of Kansas Medical Center, Mail Stop 1026, 3901 Rainbow Boulevard, Kansas City, KS 66160, USA; ^2^Department of Biostatistics, The University of Kansas Medical Center, Mail Stop 3051, 3901 Rainbow Boulevard, Kansas City, KS 66160, USA

## Abstract

*Background and Purpose*. Post-Stroke Fatigue (PSF) is a prevalent yet commonly neglected issue that impacts daily functions and quality of life in people post-stroke. To date no studies have attempted to validate a clinically-feasible and reliable instrument to quantify PSF. We developed the Visual Analog Fatigue Scale (VAFS) to eliminate difficulties and poor data validity in testing people post-stroke. The purpose of this study was to evaluate the reliability, responsiveness, and validity of the VAFS. *Methods*. Twenty-one people post-stroke (12 males, age  = 59.5 ± 10.3 years; time post-stroke  = 4.1 ± 3.5 years) participated. Subjects underwent a standardized fatigue-inducing exercise; fatigue level was assessed at rest, immediately after exercise, and after recovery. The same protocol was repeated after 14 days. *Results*. ICC values for the VAFS at rest was 0.851 (CI = 95%, 0.673 ∼ 0.936, *P* < .001), immediately after exercise was 0.846 (CI = 95%, 0.663 ∼ 0.934, *P* < .001), and 15 minutes after exercise was 0.888 (CI = 95%, 0.749 ∼ 0.953, *P* < .001). The ES values for at-rest to post-exercise and for post-exercise to post-recovery were 14.512 and 0.685, respectively. Using paired t-test, significant difference was found between VAFS scores at-rest and post-exercise (*P* < .001), and between post-exercise and post-recovery (*P* < .001). *Conclusion*. Our data suggests good reliability, responsiveness, and validity of the VAFS to assess exertion fatigue in people post-stroke.

## 1. Introduction

Post-stroke fatigue (PSF) is a prevalent symptom among survivors of stroke with an incidence rate as high as nearly 70% [1, 2]. PSF negatively affects the performance of activities of daily living [3] and may limit participation in a rehabilitation program; however PSF remains a neglected issue by clinicians and caregivers [1].

Defining and measuring fatigue is challenging as it is a multifactorial phenomenon that involves both physiological and psychosocial properties [4–6]. Fatigue that is experienced after physical exertion or use of mental effort has been defined as “exertion fatigue [7–9]”, which is characterized by being acute in nature with a rapid onset and recover period. Alternatively, “chronic fatigue” is defined as a state of weariness unrelated to previous exertion levels that is associated with prolonged stress or pathologies [8, 9]. It is important for health professionals to understand that exertion fatigue and chronic fatigue are two very different entities in order to implement appropriate therapeutic countermeasures accordingly.

Several unidimensional [7, 10–13] and multidimensional [14–17] questionnaires and scales have been developed to assess fatigue; and the appropriateness of a fatigue scale should be determined by aspects of fatigue that are being measured [18]. In people post stroke, the most commonly used scales are the Fatigue Severity Scale (FSS) [1, 2, 6, 19, 20] and the Visual Analog Scale (VAS). Although VAS was used to assess PSF in these studies [6, 19, 21], the reliability and the validity of VAS were not reported; and the investigations did not focus on identifying or differentiating different types of PSF (i.e., exertion or chronic).

To date, no studies have attempted to differentiate different types of PSF using a simple, reliable, and quantifiable measure in people with chronic stroke. We developed the Visual Analog Fatigue Scale (VAFS) as a vertical scale to eliminate difficulties and poor data validity due to visual field defect, neglect, or other visual-perceptual deficits commonly experienced among people following stroke. Thus the purpose of this preliminary study was to evaluate the reliability, responsiveness, and validity of the VAFS to assess PSF in people with chronic stroke at rest and following exercise. 

We hypothesized that VAFS will show (1) good intra rater reliability at rest, immediately post exercise, and 15 minutes post exercise, (2) a significant effect size of VAFS score change before and immediately after exercise, and (3) a direct positive relationship with physiological measures (such as heart rate and systolic blood pressure) and with the Rate of Perceived Exertion Scale (RPE).

## 2. Methods

### 2.1. Experimental Design

This study used a sample of convenience to determine intra rater reliability and validity of the VAFS in people with chronic stroke.

### 2.2. Participants

Twenty-one subjects participated in the study. Individuals were recruited from local stroke support groups and the ASTRA (Advancing Stroke Treatment through Research Alliances) participant database. Twenty-eight people from the local stroke support groups and 72 people from the ASTRA database were contacted by in-person visits and by phone, respectively. Out of the total of 100 people approached, 21 people agreed to participate and all completed the study.

To be included in this study, all participants must (1) have a diagnosis of stroke ≥6 months and ≤5 years ago, (2) have the ability to perform the exercise movement on a total-body recumbent stepper, (3) receive medical clearance from their primary care physician to confirm that the subject is medically stable and able to participate in exercise, and (4) score <2 on a dementia screening tool, the AD8 [22]. Subjects were excluded from the study if they presented with any of the following:

- hospitalization for myocardial infarction, heart surgery, or congestive heart failure during the preceding 3 months,
- recent symptoms of chest discomfort,
- resting blood pressure of 160/100 or greater,
- currently using a pacemaker,
- currently smoking or significant pulmonary pathology,
- alcoholism or alcohol dependency,
- recreational drug use,
- medication change within the duration of the study (e.g., antidepressants, cardiac medications).

The Human Subjects Committee at the University of Kansas Medical Center approved the study. Institutionally approved informed consent was obtained in writing prior to participation in the study.

### 2.3. Procedure

The first visit involved fatigue-inducing exercise and VAFS at 3 different time points. Prior to exercise, subjects were presented with the VAFS to measure fatigue at rest (VAFS_at rest1_). We then assessed the motor control capability and the severity of depressive symptoms using the Fugl-Meyer Test (FM) and Geriatric Depression Scale (GDS), respectively. Next, subjects were asked to perform a 15-minute standardized fatigue-inducing exercise. During exercise, heart rate, blood pressure, and the Rate of Perceived Exertion (RPE) [23] were recorded every 5 minutes. Immediately following the exercise, the VAFS was again administered (VAFS_post exercise1_). Subjects were allowed 15 minutes for recovery, then they were presented with the VAFS for the third time (VAFS_post recovery1_).

A second visit was scheduled 14 days after the first visit. Best effort was given to keep exercise testing and data collection at the same or similar time of the day as the first visit. The actual sequence of the second visit was identical to the first visit. Fatigue scores were assessed at rest, immediately after exercise, and 15 minutes after exercise (VAFS_at rest2_, VAFS_post exercise2_, VAFS_post recovery2_, resp.)

**Visual Analog Fatigue Scale (VAFS)**

**Figure 1:**
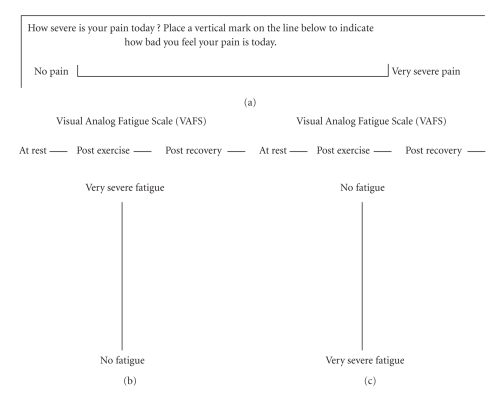
Illustration of (a) traditional visual analog scale used to measure pain, (b) VAFS version 1, and (c) VAFS version 2. VAFS: Visual Analog Fatigue Scale.

Traditionally, a visual analog scale VAS [24, 25] consists of a 10 cm horizontal line with written descriptions at each end; subjects are asked to mark on the line the point that they feel represents their perception of their current state (Figure 1). The possible score ranges from 0 to 100, measured in millimeters on a 10 cm vertical line using a pen. The score was obtained by measuring the line from “No Fatigue” to the point indicated by the subject that represents their fatigue level, the higher the VAFS score, the higher the fatigue. To avoid bias associated with the position of the text description on the VAFS (e.g., top is higher), two different versions were administered in random order. Version 1 indicated “Very Severe Fatigue” on top of the 10 cm line and “No Fatigue” below the same vertical line; while Version 2 had an opposite arrangement to represent the perceptions (Figure 1). The same version of VAFS was used at 3 different time points on the same day; subjects who received Version 1 on the first visit were administered Version 2 on the second visit.

**Fatigue-Inducing Exercise**

To induce EF, subjects were asked to perform a 15-minute standardized exercise protocol on a total-body recumbent stepper (NuStep, Inc; 5111 Venture Drive Suite 1, Ann Arbor, MI 48108). To standardize the workload, all subjects were asked to step at 75 step per minute (SPM) with an external power of 75–80 Watts (W) for 15 minutes. The device and workload of our fatigue-inducing protocol [26] were chosen to allow subjects to become safely fatigued at 40–70% [27].

**Physiologic Responses**

Heart rate and blood pressure were measured at rest and every 5 minutes during exercise. Heart rate increase (HRI) and systolic blood pressure increase (SBPI) were calculated and used for analysis.

**Rate of Perceived Exertion (RPE)**

The RPE [23] was used to determine the highest level of effort exerted perceived by subjects. It was recorded every 5 minutes during the fatigue-inducing exercise.

**Fugl-Meyer (FM)**

The FM was used to determine the level of motor function in the hemiparetic limbs following stroke. The FM is a reliable and valid tool that was specifically designed as a clinical measure of sensorimotor impairment for stroke [28]. The total possible score is 124, which consists of sensation (FMSEN-24), upper extremity (FMUE-66), and lower extremity (FMLE-34). Because the fatigue-inducing exercise in this study required motor performance of all four limbs, the combined total-motor (FMTM) scores of the FMUE and FMLE were determined (FMTM = FMUE + FMLE). The possible score ranges from 0 to 100.

**Geriatric Depression Scale (GDS)**

The GDS is a questionnaire that includes 30 items that refer to affective, cognitive, and behavioral symptoms of depression to assess mood. It has been tested and used extensively with the older population with good validity and reliability [29]. The possible score ranges from 0 to 30.

### 2.4. Data Analysis

SPSS 15.0 (SPSS, Inc; 233 S. Wacker Drive 11th Floor Chicago, IL 60606) statistical software was used to perform all statistical analysis; alpha level of 0.05 was used to determine statistical significance. Descriptive statistics were calculated. EF was calculated by subtracting VAFS_at rest_ score from VAFS_post exercise_ score (VAFS_post exercise_ − VAFS_at rest_). Recovery Rate (RR) was calculated as the percentage using the formula: (VAFS_post exercise_−VAFS_post recovery_)/(VAFS_post exercise_ − VAFS_at rest_) × 100.

**Reliability**

The intraclass correlation coefficient (ICC_3,1_) was calculated to determine intra rater reliability of repeated measures taken during Visits 1 and 2 (i.e.,  VAFS_at rest1_ versus VAFS_at rest2_, VAFS_post exercise1_ versus VAFS_post exercise2_, VAFS_post recovery1_ versus VAFS_post recovery2_, EF_1_ versus EF_2_, and RR_1_ versus RR_2_). Test-retest reliability was assessed using the Bland-Altman plot by examining the shift in the measurement and the variability in the VAFS change score.

**Responsiveness**

To examine the ability of the VAFS to detect change over time, the Effect Size (ES) was calculated as an index of responsiveness using the formula ES = mean (VAFS_post exercise_ − VAFS_at rest_)/standard deviation VAFS_post exercise_ [30]. In addition, paired *t*-test was used to determine difference between scores at rest and post exercise, and between post exercise and post recovery.

**Validity**

The peak RPE values were used for analysis; heart rate increase (HRI) and systolic blood pressure increase (SBPI) were calculated by subtracting respective values at rest from the peak values during the exercise. Pearson's correlation coefficient was used to determine the relationship between EF and HRI, EF and SBPI, and EF and RPE.

## 3. Results

Characteristics of the 21 subjects are shown in Table 1.

### 3.1. Reliability of the VAFS

**Figure 2:**
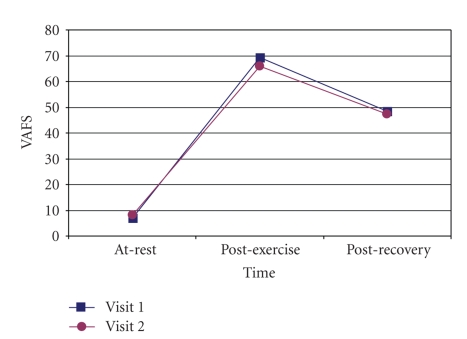
VAFS measures at rest, post exercise, and post recovery from Visits 1 and 2. VAFS: Visual Analog Fatigue Scale.

**Figure 3:**
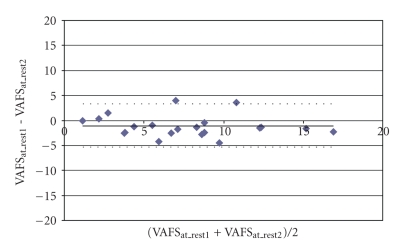
Bland-Altman plot of the VAFS scores at rest from Visits 1 and 2 indicates no shift and low variability. VAFS: Visual Analog Fatigue Scale.

Good [31] intra rater reliability was found for the VAFS measures taken during the 2 separate sessions. ICC values for the VAFS at rest were 0.851 (CI = 95%, 0.673~0.936, *P* < .001), immediately after exercise were (CI = 95%, 0.663~0.934, *P* < .001), and 15 minutes after exercise were 0.888 (CI = 95%, 0.749~0.953, *P* < .001) as shown in Table 2. The VAFS measures of both visits are illustrated in Figure 2; the Bland-Altman plot in Figure 3 illustrates the presence of a shift in the VAFS measure at rest, with relatively low variability. In addition, the intra rater reliability for the EF and the RR was also good [31] with the ICC values of 0.829 (CI = 95%, 0.631~0.927, *P* < .001) and 0.893 (CI = 95%, 0.760~0.955, *P* < .001), respectively.

### 3.2. Responsiveness

The ES values for at rest to post exercise and for post exercise to post recovery were 14.512 and 0.685, respectively. A large ES was found for the change in VAFS scores between at rest and immediately post exercise; and a moderate ES was found for the change between immediately post exercise and post recovery [30]. Using the paired *t*-test, significant difference was found between VAFS scores at rest and post exercise (*P* < .001), and between post exercise and post recovery (*P* < .001).

### 3.3. Validity Testing

**Figure 4:**
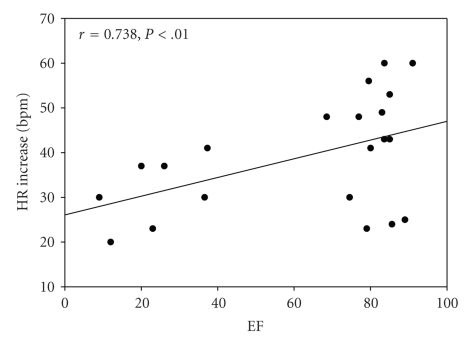
Scatterplot illustrating the relationship between EF and HR increase. EF: Exertion Fatigue, HR: Heart rate, and BPM: beats per minute.

**Figure 5:**
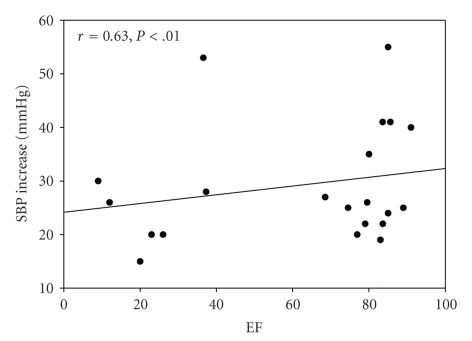
Scatterplot illustrating the relationship between EF and SBP increase. EF: Exertion Fatigue, SBP: Systolic Blood Pressure, and mmHg: Millimeter of mercury.

HR increase was 41.1 (±14.9) beats per minute, SBP increase was 37.1 (±14.0) mmHg, and the RPE was 15.8 (±2.9). A significant positive relationship was found using Pearson's correlation coefficient for EF and HRI (*r* = 0.738; *P* = .00), EF and SBPI (*r* = 0.630; *P* = .02), and EF and RPE (*r* = 0.802; *P* = .00). The relationship between the EF, HR, and SBP is illustrated in Figures 4 and 5, respectively.

## 4. Discussion

Our preliminary data demonstrated good intra rater reliability of the VAFS at rest, immediately after exercise, and 15 minutes post exercise. This finding suggests that the VAFS may be a reliable instrument to assess PSF in people post stroke. The VAFS also demonstrated good intra rater reliability to measure exertion fatigue (EF) and recovery rate (RR).

The Visual Analogue Scale is commonly used to measure pain in clinical studies. For example, a recent study that examined the validity and reliability of the Visual Analogue Scale in measuring acute abdominal pain found a high intraclass correlation coefficient (ICC = 0.990) [32].

This finding is consistent with the current study to suggest good test-retest reliability for the measures taken at separate times. However, several fatigue studies that utilized the original Visual Analogue Scale to assess PSF did not establish reliability or validity and used a simple measure to describe a complicated, poorly-defined phenomenon [6, 19, 21]. Unlike chronic fatigue, in order to accurately measure exertion fatigue, the VAFS must be administered at the appropriate time; and to calculate exertion fatigue, values need to be determined by subtracting the VAFS score at baseline from the score assessed immediately after exercise. 

The VAFS demonstrated large responsiveness to changes between at rest and immediately after exercise, and moderate responsiveness between immediately after exercise and 15 minutes post exercise, as determined by the Effect Size. An Effect Size value of 0.4 is considered small, 0.5 is considered moderate, and 0.8 and greater is viewed as large [30]. Our analysis was designed to examine the ability of the VAFS to detect an immediate change of fatigue level due to exercise over a short period of time; changes of long-term fatigue level due to exercise training effect over a prolonged time period were not intended as part of our analyses.

Our data showed a significant positive relationship between the fatigue induced by exercise (measured by EF) and physiologic responses (i.e., heart rate and blood pressure, measured by HRI and SBPI, resp.). We also found a significant positive relationship between EF and subjectively perceived level of exertion (measured by RPE). The RPE and VAFS measure different constructs, as the RPE reflects the perceived level of exertion during exercise, not the level of exertion fatigue after exercise. In addition, by using VAFS, subjects are required to base their answers on intuitive response at the moment, which may help avoid recollections of previous references that are verbal or numerical. These findings indicate that VAFS is a valid instrument to measure fatigue following exercise in people with chronic stroke. 

Consistent with the findings of Choi-Kwan and colleagues [19], we found a high occurrence of fatigue in people post stroke. Using the VAFS, we found evidence of baseline fatigue in most subjects (20 out of 21) at rest prior to fatigue-inducing exercise. This discovery indicates that this type of baseline fatigue can exist independently of physical stress, which supports previous findings [33] to suggest a distinct construct in PSF that is persistent and chronic in nature. In 2002, Glader and colleagues conducted a follow-up study to investigate PSF in people 2 years post stroke and concluded that PSF is frequent and can be persistent and chronic even late after stroke [33]. 

One of the limitations in this pilot study is the small sample size recruited from a single center. The authors acknowledge that the small sample size from the same region may limit the generalizability of our findings. Nevertheless, the significant contribution of this pilot work is that it provides a clinically feasible tool specifically designed to assess fatigue in people post stroke. Follow-up studies should utilize a larger sample size to further solidify the validity of the VAFS scale.

While the Rate of Perceived Exertion (RPE) [34] and heart rate (HR) can reflect the intensity of an exercise or activity, they do not detect the level of fatigue. Although related, the level of exertion (or intensity, as represented by HR) and level of fatigue are two distinct measures. For example, an individual can exert at a high intensity level (high RPE at the moment) but only experience moderate or even low level of fatigue. In order to faithfully report fatigue that is induced by exercise, exertion fatigue must be calculated by determining the difference between fatigue level at baseline and immediately after exercise. To the authors' understanding, previous fatigue assessments and questionnaires measure only fatigue level over an extended period of time (e.g., two weeks), instead of measuring “changes” that are brought on by a specific event. In addition, most investigators used the visual analog assessment at a single time point to represent fatigue; our fatigue measurement was designed to quantify changes of fatigue level in real time. 

Previous study has shown that up to 40% of people post stroke can have depressive symptoms [1]. We did not screen for depression but measured severity of depressive symptoms in this study. Although it has been suggested that depression may be closely related to fatigue [2, 5], previous studies used questionnaires that focused on fatigue level that is chronic in nature over a two-week period, which may not be the same type of fatigue that is brought on by exercise as induced in our study. Our data suggest that the VAFS show promising responsiveness to detect changes of fatigue level in real time.

Although statistical significance was achieved, we were not able to do subgroup analyses to determine whether reliability and validity of VAFS would be different between gender, different types of stroke, and different times post stroke. In addition, although baseline fatigue was detected, this study was not designed to distinguish other types of fatigue such as chronic fatigue or circadian fatigue. Future studies may also consider measuring fatigue at different times of the day in order to detect such matters. 

Our data suggest that exertion fatigue may be detected in real-time fashion using the VAFS, which is unique from the type of fatigue that is chronic in nature and is measured by questionnaires that focus on the past 2–4 weeks. Future studies should investigate on differentiating other types of fatigue using appropriate measurement that is sensitive to the nature of the fatigue construct.

Finally, future studies need to identify predictors of PSF in order to address fatigue issues with an intervention in people post stroke.

## 5. Conclusion

In conclusion, although the psychometric properties of the VAFS have not yet been thoroughly evaluated and the data should be interpreted with caution, our preliminary finding suggests that the reliability, responsiveness, and validity of the Visual Analog Fatigue Scale (VAFS) appear to be promising to assess exertion fatigue in people with chronic stroke.

## Figures and Tables

**Table 1:** Participant characteristics (*n* = 21). Values are means ± SD.

Characteristics	Values
Men/women	12/9
Stroke lesion side: right/left/brain stem	15/4/2
Stroke subtype: ischemic/hemorrhagic	18/3
Age (years)	59.5 ± 10.3
Time post stroke (years)	4.1 ± 3.5
FMTM	70.8 ± 28.8
GDS	10.2 ± 7.3

FMTM: Fugl-Meyer Total-Motor score.

GDS: Geriatric Depression Scale.

**Table 2:** The intraclass correlation coefficient of measures on 2 separate visits (*n* = 21).

	Visit 1	Visit 2	ICC
VAFS_at rest_	7.2 ± 4.3	8.3 ± 4.5	0.851
VAFS_post exercise_	69.4 ± 30.5	65.8 ± 31.9	0.846
VAFS_post recovery_	48.5 ± 25.4	47.5 ± 26.9	0.888
EF	62.4 ± 29.3	57.5 ± 30.9	0.829
RR (%)	37.0 ± 17.3	37.7 ± 15.9	0.893

VAFS: Visual Analog Fatigue Scale.

EF: Exertion Fatigue (EF =VAFS_post exercise_−VAFS_at rest_).

RR: Recovery Rate (RR = VAFS_post exercise_− VAFS_post recovery_)/(VAFS_post exercise_−VAFS_at rest_) × 100.

ICC: Intraclass correlation coefficient, model (3,1).
